# A Balanced Arthroscopic Debridement of the Inner Layer of the Knee Retinaculum Increases the Tibiofemoral Joint Space Width

**DOI:** 10.1155/2022/1766401

**Published:** 2022-01-29

**Authors:** Roberto Yáñez-Diaz, Lars Strömbäck, Francisco Vergara, Gaston Caracciolo, Anthony Saravia, Carlos Sandoval, Héctor Zamorano, Sebastián Abusleme, Carlos De la Fuente

**Affiliations:** ^1^Traumatologia, Clínica MEDS, Santiago, Chile; ^2^Centro de Innovación, Clínica MEDS, Santiago, Chile; ^3^Carrera de Kinesiología, Departamento de Cs. de La Salud, Facultad de Medicina, Pontificia Universidad Católica, Santiago, Chile; ^4^Laboratory of Neuromechanics, Universidade Federal Do Pampa, Campus Uruguaiana, Uruguaiana, Brazil

## Abstract

**Introduction:**

Traditional techniques can enlarge the medial tibiofemoral joint space width (JSW) for meniscal repairs, but a remnant ligament laxity may be developed. Alternatively, the debridement of the inner retinaculum layer may result in a balanced JSW without causing extra-ligament damage (retinaculum layers II and collateral ligament).

**Purpose:**

The purpose of this study was to determine whether a concentric arthroscopic debridement of the inner retinaculum layer increases the tibiofemoral JSW in patients with meniscal injuries. Secondarily, we determine whether the increase in JSW is symmetrical between compartments and describe the rate of complications and patient satisfaction.

**Method:**

Twenty middle-aged (15 male and five female) patients diagnosed with acute meniscal injury aged 36 ± 12 years were enrolled. The patients were submitted to an arthroscopic debridement of the inner layer of the knee retinaculum for both the medial and lateral compartments. The tibiofemoral JSW was measured intra-articularly using a custom instrument. A two-way ANOVA for repeated measures was used to compare the JSW. A Bland–Altman analysis and test-retest analysis were performed.

**Results:**

The JSW increased following the debridement of the inner retinaculum layer, for both the medial and lateral compartments (*p* < 0.001). No complications were identified, and the patients were satisfied with the intervention. The minimal detectable change and bias of the custom instrument were 0.06 mm and 0.02 mm, respectively.

**Conclusion:**

The debridement allows a clinically important (>1 mm) symmetric tibiofemoral JSW enlargement. The technique suggests favoring the diagnosis of meniscus injuries and manipulating arthroscopic instruments without secondary complications after one year.

## 1. Introduction

Diminishing or avoiding knee factors that predispose to degenerative joint disease following meniscus injuries is one of the most important clinical concerns [1]. A significant increase in tibiofemoral peak contact pressure occurs after meniscal injuries and can lead to cartilage degeneration [2]. For instance, a knee with total or partial meniscectomy during knee flexion from 0° to 60° led to an increase in intra-articular pressure to values higher than 40% compared with the repaired meniscus [3]. Therefore, there is a broad consensus to maximize the effort to restore the anatomy of the meniscus when possible [4]. However, a complication related to a lack of tibiofemoral joint space width (JSW) for the arthroscopy repair may cause undesired cartilage damage when visualizing the posterior structures [5].

The pie crust technique is a common intervention among surgeons to improve the visualization of the meniscus [6] and has been indicated to access the posterior medial meniscal horn, visually diagnose the meniscal tears, and generate less risk of an iatrogenic cartilage lesion [7]. The technique applies tension to the medial collateral ligament and valgus stress after repeated perforations of the capsuloligamentous structures to enlarge the medial JSW, resulting in a medial compartment opening of between 3 and 5 mm [8]. Unfortunately, the pie crust technique lengthens the entire layers of the retinaculum and medial collateral ligament [9], which can cause an increase in valgus, internal rotation, posterior drawer in extension, and anterior tibial drawer of the flexed and externally rotated knee [10]. The involved damage of the ligaments can also compromise the sensorimotor stabilization of the frontal knee plane [11]. Besides, the technique appears to be mechanically unbalanced between compartments, and unfortunately, a remnant medial laxity can also compromise the contact forces on the cartilage and meniscus, generating stress concentration [12].

The deepest layer of the retinaculum is externally surrounded by fat tissue and is known as the inner retinaculum layer (femoral meniscal ligament, tibial meniscal ligament, and the third layer of the lateral retinaculum). This layer does not have a relevant mechanical role in patellofemoral stabilization in comparison with the fibrous expansions of the longitudinal and obliquus vastus medialis, medial patellofemoral ligament (transverse fibers), medial patellofemoral ligament transverse (obliquus fibers), medial patellotibial ligament, medial patellomeniscal ligament, fascia lata, crural fascia, fibrous expansions of the vastus lateralis, superficial oblique retinaculum, patellotibial band, epicondylopatellar band, deep transverse retinaculum, iliotibial band extension to the patella, lateral patellofemoral ligament, lateral patellotibial ligament, and lateral patellomeniscal ligament [13–27]. As the deep layer of the retinaculum is attached proximal to the femoral and tibial joint margins, its debridement may lead to a balanced opening of the tibiofemoral join space, similar to the aim of the pie crust technique, but without causing damage to layers II and III of the retinaculum and collateral ligaments. This technique may also avoid compromising the contact forces on the cartilage and meniscus. Therefore, the purpose of this study was to determine whether a concentric arthroscopic debridement of the inner retinaculum layer increases the tibiofemoral JSW in patients with meniscal injuries. Secondarily, we determine whether the increase in JSW is symmetrical between compartments, describe the rate of complications and patient satisfaction, and assess the reproducibility of a custom device to measure the JSW.

## 2. Materials and Methods

### 2.1. Study Design

We conducted a prospective comparative study, prior to and following an arthroscopic debridement of the inner layer of the knee retinaculum, of both the medial and lateral compartments. Participants were middle-aged men and women who suffered a sports injury and were recruited from the MEDS Clinic during 2019. Each participant provided written consent to be included in the study and to be submitted to the surgery. This study was approved by the ethics committee of the local institution and was developed following the Declaration of Helsinki.

### 2.2. Participants

Twenty middle-aged (15 male and five female) patients diagnosed with acute meniscal injury (age of 36 ± 12 years, height and time of evolution of 11.3 ± 1.3 months) were enrolled. The meniscus tear characteristics are summarized in Table 1.

Sample size estimation resulted in 20 patients with a sample size of 0.35. The estimation was made using five patients who were excluded from the study. For the estimation, a mixed ANOVA of repeated measures with an alpha error of 5% and a statistical power of 80%, two groups, and time repetition a priori were used to perform the calculations using the G Power software 3.1.9.2 (Düsseldorf Universität, Germany).

The inclusion criteria for patients were (i) men aged between 18 and 55 years, (ii) competitive sports practice during the weekends, (iii) acute meniscal injury (no more than 30 days of rupture), and (iv) first injury. The exclusions criteria were (i) ligament injury, (ii) laxity, (iii) revision surgeries, (iv) collagen pathology, (v) history of autoimmune diseases, (vi) prior history of a traumatic lower leg injury, (iv) orthopedic alteration of the lower leg, and (v) steroid therapy dependence.

### 2.3. Surgery

**Figure 1:**
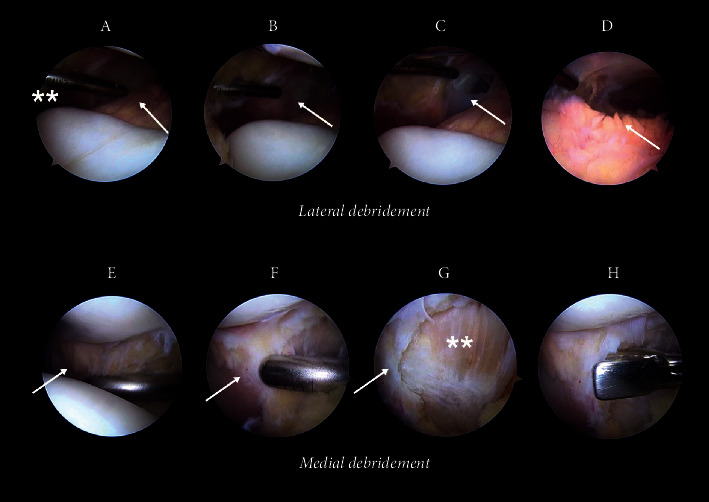
Concentric arthroscopic debridement of the inner reticulum. (a) A to D show a progressive sequence of the lateral debridement of the inner medial retinaculum. E to H show a progressive sequence of the medial debridement of the inner medial retinaculum. The asterisks show the lateral portal location, and the arrow shows the inner retinaculum. (b) The image shows the distal debridement, and the arrow shows the inner retinaculum. (c) The image shows the intact layer II of the retinaculum, and the arrow shows the inner layer of the retinaculum. (d) The image shows fibrin in the meniscus, and adherences are visualized at the inner layer of the retinaculum. (e) The image shows the debrided inner medial layer of the retinaculum, and the arrow shows the intact inner layer. (f) The image shows the inner medial layer of the retinaculum under debridement, and the arrow shows the intact inner layer. (g) The asterisks show the medial layer II of the retinaculum, and the arrow shows the inner layer. (h) The debridement is completed using a basket punch.

Following a traditional diagnostic arthroscopy of the knee [28], a standard shaver (Excalibur Blade, Arthrex, Inc., USA) Ø 4.0 mm in oscillate mode with suction was used to debride the deep layer of the retinaculum through traditional anteromedial and anterolateral portals with the knee in extension, as shown in Figure 1. The shaver was placed in the anteromedial portal, while the optical unit was introduced via the anterolateral portal to debride the inner lateral retinaculum. Partial resection of the Hoffa fat was performed to improve the visualization of the compartments beginning with the portal. The shaver should be moved over the inner retinaculum with an intermittent radial movement along a radius that begins in the portal and ends at the site of the debridement in a “windscreen movement,” displacing the shaver from the portal to the posterior compartment of the knee surrounding the retinaculum and ending with a displacement to the middle line of the knee. The same procedure is repeated for the contralateral portal. The shaver should never be static to avoid ligamentous damage, for example, by debriding layer II of the retinaculum. When debridement ends, the shaver is shifted into the anterolateral portal and the optical unit is introduced via the anteromedial portal to debride the deep medial layer of the retinaculum. Typically, the medial layer is thicker than the lateral layer. Hence, when the lateral layer is debrided, it is possible to obtain two sublayers where the last layer can be debrided using a basket punch. Vascular roots of the geniculate vessels should not be visible after the debridement, but if the vascular vessels are visible, it is recommendable to coagulate the capsular vessels with radiofrequency (ApolloRF, Arthrex, Inc., USA). The average duration of the procedure is ∼3 to 5 min, and we used the same rehabilitation management as is used for traditional meniscal repairs, which lasts ∼3 months post-surgery; the return to sport is expected to start during this period in some patients. However, according to a systematic meta-analysis of 453 studies, the average rehabilitation period was 5.6 months, with a range of 3 to 8 months [29].

All surgical procedures were performed by the same senior surgeon (RY) who has more than 20 years of experience in arthroscopic meniscal repairs and by a second assistant surgeon (LS, FV, GC, AS, CS, HZ, or SA). The second surgeon was one of the six specialized arthroscopy knee surgeons who were part of this study. The inclusion criteria for the surgeons in this study were (i) specialized arthroscopic knee surgeons from the MEDS Clinic, (ii) fellowship in knee surgery for at least one year, (iii) professional experience as a specialist knee surgeon for at least 5 years, and (iv) excellent follow-up of their operated patients. The exclusion criteria were (i) no experience with meniscal repairs, (ii) not familiarized with the protocol of the study, (iii) history of repetitive surgery failures, and (iv) poor ability with arthroscopy instruments.

### 2.4. Custom Instrument for the Measurement of the JSW

**Figure 2:**
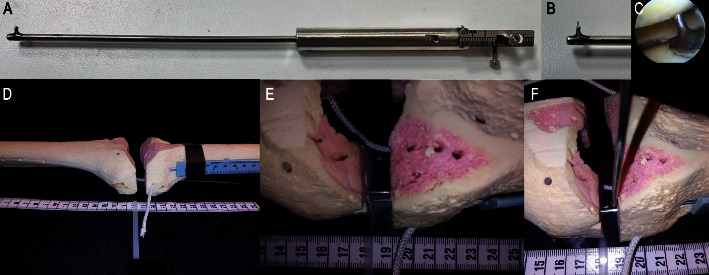
Custom instrument to measure the tibiofemoral joint space width using osseous phantoms for practicing. (a) Custom instrument. (b) Distal end of the instrument. (c) Arthroscopic measurement after debridement of a patient. (d) Setup for custom instrument analysis. A Kirschner wire linked the tibia to the femur, controlling the vertical tibial displacement. (e) A Vernier caliper was introduced vertically into the tibial phantom to measure different joint space widths. (f) Custom instrument used to measure the tibiofemoral joint space width, while the Vernier caliper also measures the same width for the Bland–Altman and test-retest analyses.

The authors created a cylindrical telescopic instrument (length of 23 cm, distal diameter of 1.5 mm) capable of being introduced into the knee through the arthroscopic portals (Figure 2). The instrument had a thin circular filament (0.02 mm in diameter with a maximal distal length of 3 mm) capable of sliding towards the tibiofemoral space permitting the vertical measurement of the JSW at the end of the instrument (Figure 2). The resolution of the device was 0.1 mm and had a vertical range of measurement between 1.5 and 7.5 mm. The instrument was made of surgical stainless steel.

The instrument was compared to a standardized digital Vernier caliper with a resolution of 0.05 mm (Mitutoyo, Japan). The caliper was fixed in a tibial phantom, permitting to measure different JSWs (Figure 2). The measurements of the JSW varied from 0 to 6 mm, each 0.05 mm, with the testing repeated three times.

### 2.5. Outcomes

The tibiofemoral JSW corresponded to the vertical length between the tibial and the femoral cartilage measured at the lowest JSW for each compartment [30]. The variable was directly measured in millimeters using the custom device previously described. The rate of complications is given as a percentage, and the level of patient satisfaction was recorded as satisfied or not satisfied.

### 2.6. Statistical Analysis

Data were reported as mean, standard deviation, and 95% confidence interval. A two-way ANOVA for repeated measures in a mixed model comparing the tibiofemoral JSW was performed, with two factors: two (time) × two (compartments) and an alpha of 5%. The sphericity of data was corrected using the Greenhouse–Geisser criteria (*p* < 0.001). A post hoc analysis of pairwise comparisons was performed using the Bonferroni test with an alpha of 5%. A Bland–Altman analysis was performed to study the degree of agreement between the instruments (limits of agreement, correlation, R-square, average absolute bias, and minimal detectable change). The test-retest reliability was also obtained using Cronbach's alpha. All statistical analyses were performed using SPSS® (IBM, USA).

## 3. Theory

Arthroscopy debridement is a technique where selected structures of the knee are contoured or removed mechanically through small knee portals with modern instrumentation [31]. The selection of the shaver, velocity, irrigation, suction, and magnitude of the debridement is crucial for the development of an anatomic procedure [31–33]. The risks may include infection, bleeding, injury, formation of a blood clot, and nerve or blood vessel damage and stiffness [31].

## 4. Results

**Figure 3:**
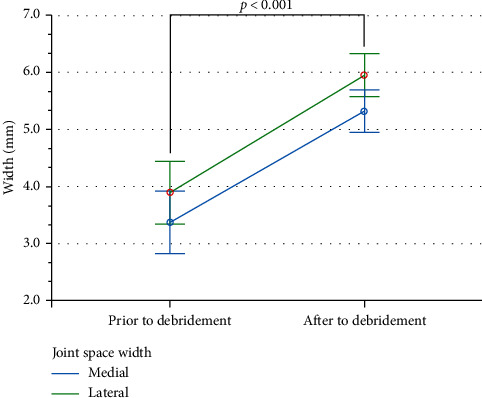
Tibiofemoral joint space width increase following the arthroscopic debridement of the inner retinaculum layer. The error bars correspond to the 95% confidence interval.

The tibiofemoral JSW increased following the arthroscopic debridement of the inner layer of the retinaculum of the knee, in both the medial and lateral compartments (Table 1 and Figure 3). In this study, there was a main effect of time (*F*(1,38) = 211.3, *p* < 0.001, ŋ2 = 0.854), but not for compartments (*F*(1, 38) = 3.801, *p* = 0.059, ŋ2 = 0.096), and there was no interaction (*F*(1,38) = 0.146, *p* = 0.704, ŋ2 = 0.004). The patients did not develop acute complications, with 5% describing patellofemoral pain (1/20) and meniscus re-rupture (1/20) 1 year after and 10% describing inter-joint line pain (2/20). All patients reported the highest level of satisfaction at 1-year follow-up (Table 2).

The Bland–Altman analysis showed limits of agreement between −0.21 mm and 0.94 mm, an average absolute bias of 0.02 mm, a minimal detectable change of 0.06 mm, a correlation between measurements of 0.99, and an R-square of 0.97. Cronbach's alpha was 0.99.

## 5. Discussion

The main findings of this study are that a concentric arthroscopic debridement of the inner layer of the knee retinaculum prior to a meniscal repair results in a balanced increase in the tibiofemoral JSW, without an increase in the rate of complications, and high levels of patient satisfaction 1 year after the intervention. These findings are clinically relevant for knee surgery, because this allows surgeons to enlarge the JSW for arthroscopic instrument manipulation with low risk of cartilage damage, i.e., for repairing the posterior meniscal horns. Additionally, the procedure improves the visual access for better diagnosis of possible damage of the intra-articular structures, improving intraoperative decision-making and potentially reducing the rate of false-negative injuries. Also, the procedure is unlikely to affect the joint instability provoked by damage to the ligaments of layer II (involved mechanically in the control of the patellofemoral or tibiofemoral joints), because the intervention is aimed at the deepest ligaments of the knee retinaculum.

The controlled debridement of the inner layer of the retinaculum permits a clinically important JSW increase (>1 mm), as we have shown here. The JSW increase is likely because the inner retinaculum layer is shorter than its external layers, allowing physiological retinaculum deformation under low-acting force (toe phase of the force-length ligament curve or slack ligament) [34]. Hence, this debridement increases the JSW sufficiently to allow better arthroscopy instrument manipulation in the knee. However, care must be taken to avoid technical error leading to excessive debridement, releasing layer II of the patellofemoral retinaculum, and damaging unnecessary ligamentous and vascular-nervous structures. The most likely potential acute errors are the debridement of external layers affecting the patellofemoral joint, the damage of geniculate vessels, and excessive debridement of neural ends with consequent excessive hemarthrosis and paresthesia, which may lead to the need for evacuations of the hematoma and cauterization [35]. The potential long-term effects of excessive debridement include weakening of the extensor function of the knee, patellofemoral instability and pain, and knee laxity [36]. The authors recommend taking guidance based on the fat tissue surrounding the inner layer of the retinaculum, and the ligamentous layer immediately external to the fat tissue may be a useful anatomical mark of the limits of debridement. Also, special consideration should be given to adhesion formation due to intra-articular bleeding [37], which we have usually seen attached from the meniscus to the joint capsule, requiring identification and debridement.

An unbalanced debridement of the knee ligaments in the frontal plane has been indicated to concentrate the stress on the cartilage and had been shown to generate osteoarthritic changes in animal models [38]. Hence, the control of the symmetrical enlargement of the JSW between compartments by the surgeon is crucial when the JSW is targeted. Our study did not find an asymmetrical JSW increase (medial: 1.9 mm vs. lateral: 2.1 mm), and the findings suggest that the intervention does not cause unbalanced results. In contrast, the pie crust technique, which aims to achieve a similar objective of JSW increase, appears to lengthen the knee joint through unbalanced ligament lengthening, which favors the apparition of a remnant medial laxity in patients at least six months post-surgery, adding secondary biomechanical complications to the rehabilitation [9, 12].

In our study, the intervention did not lead to acute complications such as adverse events, joint or wound infections, infectious complications, thromboembolic events, or mortality. The post-surgery care must be closely supervised by the healthcare team, including medical doctors, nurses, and physical therapists, to keep the rate of complications low as seen in this study. On the other hand, one of the 20 patients described patellofemoral pain after one year (5%). This patient appears to have been more likely to develop patellofemoral joint overload due to a secondary quadriceps weakness after checking their medical records regarding the pain. In summary, the findings suggest that the intervention does not increase the rate of complications after knee arthroscopy, which is around 1 to 5% [39], and the patient perceptions of the intervention were satisfactory.

The custom instrument showed a high JSW measurement accuracy compared with a standard instrument. Remarkably, the findings obtained can be attributed to the intervention, as its minimal detectable change was lower than the increase in the JSW detected after the debridement.

The main limitation of this study is related to the COVID-19 pandemic. At our center, the patients are frequently submitted to biomechanical assessment after one year, but the recruited sampled avoided this to reduce the risk of contracting COVID-19. Furthermore, the government action to prevent the spread of SARS-CoV-2 limited all clinical assessments not related to the COVID-19 pandemic. Lastly, the lower limit for the minimal measurement of our custom instrument (<1.5 mm) could be improved in the next version with new geometry.

## 6. Conclusion

The arthroscopic debridement of the inner layer of the knee retinaculum results in a clinically important (>1 mm) balanced increase in the tibiofemoral JSW without increasing the rate of complications after one year. The patients were highly satisfied one year after the intervention. The intervention is clinically relevant because it is an effective debridement method that is able to facilitate arthroscopic diagnosis and manipulation of arthroscopic instruments during knee surgery.

## Figures and Tables

**Table 1:** Descriptive statistics of joint space width, meniscal tear, and treatment.

	Without debridement	With debridement	Δ
	Mean ± Sd	Mean ± Sd	
	*n* = 20	*n* = 20	
Compartment			
Medial, mm.	3.4 ± 1.0	5.3 ± 0.7	1.9
Lateral, mm.	3.8 ± 1.4	5.9 ± 0.9	2.1
	Number of cases *n* = 20		
Meniscal tear			
Longitudinal	60% (12/20)		
Radial	5% (1/20)		
Horizontal	0% (0/20)		
Bucket handle	20% (4/20)		
Parrot beak	0% (0/20)		
Flap	5% (1/20)		
Mixed	10% (2/20)		
Meniscal repair			
Meniscal suturing	10% (2/20)		
Partial meniscectomy	90% (18/20)		
Total meniscectomy	0% (0/20)		

Sd : standard deviation. Δ = differences between compartment spaces without and with debridement.

**Table 2:** Complications, symptoms, and satisfaction following 1 year of the intervention.

	Cases	
	% (proportion)	
	*n* = 20	
Acute (first 10 days of) complications		
Any adverse event	0% (0/20)	
Joint infection	0% (0/20)	
Wound infection	0% (0/20)	
Infectious complications	0% (0/20)	
Thromboembolic event	0% (0/20)	
Mortality	0% (0/20)	

	Cases	
	% (proportion)	
	*n* = 20	
Following 1-year symptoms		
Loss of sensitivity	0% (0/20)	
Patellofemoral instability	0% (0/20)	
Patellofemoral pain	5% (1/20)	
Inter-joint line pain	10% (2/20)	
Tibiofemoral instability	0% (0/20)	
Meniscal re-rupture	5% (1/20)	
Cartilage damage	0% (0/20)	
Sensation of instability	0% (0/20)	

	Satisfied cases % (proportion) *n* = 20	Unsatisfied cases % (proportion) *n* = 20
Satisfaction with the surgery	100% (20/20)	0% (0/20)

## Data Availability

Data can be found in https://www.researchgate.net/publication/357835641_Supplementary_data_A_balanced_arthroscopic_debridement_of_the_inner_layer_of_the_knee_retinaculum_increases_the_tibiofemoral_joint_space_width.
